# An Important Military Research: The Control of Bilharzia Disease

**Published:** 1916-01-15

**Authors:** 


### AN IMPORTANT MILITARY RESEARCH.

### The Control of Bilharzia Disease.

The war has had one result, at least, which will prove a blessing and a benefit, unless we are greatly mistaken, to humanity at large. We refer to the unravelling* of the mystery which has hitherto surrounded that widely spread malady of certain tropical and sub-tropical countries known as Bilharziasis or Bilharzia disease. This unpleasant and disabling pest is pretty widely spread on the globe: it is known in Cyprus, Madagascar, Mauritius, Réunion, Mesopotamia, the West Indies, India, Australia, and South America; but its chief distribution is in North, East, and South Africa, especially in Egypt and along the Mediterranean coast. The enormous increase in the garrison of Egypt, where bilharzia disease is most common, led to fears that considerable wastage among the troops might occur from this cause. So, taking time by the forelock, the authorities dispatched a well-known expert in tropical medicine, Mr. R. T. Leiper, to Egypt, with the military rank of lieutenant-colonel, in the hopes that he might solve the problem of how the contagion is transferred; for, obviously, until this is discovered preventive measures can have no rational basis and are mere empirical groping in the dark.

### The Natural History of the Worm.

In order to appreciate the nature of the problem which Lieutenant-Colonel Leiper was asked to solve, a brief sketch of the natural history of the disease is necessary. To begin with, it can be assumed that bilharziasis has plagued the Egyptians for many centuries, for ancient mummies have been found in which the infection could be demonstrated. Larrey, Napoleon's leading surgeon, described a serious outbreak of it among the French troops in Egypt in 1799-1801. In 1851 Bilharz discovered that the disease is due to a minute worm which exists in the portal vein of infected individuals, and that the characteristic symptoms of blood in the stools and urine, pain, and loss of energy are due to the presence of the parasite's eggs in the intestines and bladder. He named the worm *distomum hæmatobium*, but did not, however, discover how it gets into the body, nor has anyone else hitherto done so. For many years it has been believed that the worms can penetrate the unbroken skin, and that they gain access to the body by attacking human beings during the act of bathing. Another view is that the drinking of infected water is the cause. Lieutenant-Colonel Leiper holds that both methods of infection are to be guarded against.

The bilharzia worm belongs to a tribe of which many members undergo a cycle of development in an "intermediate host," frequently a mollusc. That is to say, the eggs of the worm do not hatch into young worms, but into other creatures which must invade a mollusc before they in their turn can act as parents of the original worm. It was accordingly conjectured that very possibly there is some such "intermediate host" in the case of this particular member of the genus also, and many workers searched for years among the molluscs and the fresh-water fishes of Egypt, but without success. Then in 1894 Dr. Looss, of Leipzig, who had also searched in vain for the hypothetical host, advanced the opinion that no such host exists, but that direct infection from man to man can and does take place. It is true that he never proved this contention, and that several of the most eminent of tropical hygienists (Manson and others) spoke of it with great reserve; but Looss' authority was such that it was very widely accepted.

### The Incrimination of the Snail.

If Lieut.-Colonel Leiper's researches are confirmed—and they seem convincing beyond all possibility of error—these views of Dr. Looss must be abandoned finally and for ever. The host has been identified and the cycle of development traced back to the worm form. When the eggs of bilharzia escape in the excreta of an infected individual, and are favoured by due conditions of warmth and moisture, they hatch out into a minute active creature known as a *miracidium*; this is immediately attracted to certain kinds of snail, if they are present in the water, and penetrates their bodies. Within the snail it undergoes metamorphosis, and is finally the parent of a *cercaria*, and it is this which is capable of infecting human beings and starting the cycle of development all over again by growing up into a worm and laying eggs as soon as it is adult. It is not necessary to follow in detail the patient observations and ingenious experiments on which this theory is founded: suffice it that it seems quite definitely proved. The important result from the hygienist's point of view, both in regard to our troops and to the natives of Egypt, is that the preventive measures which are indicated in these circumstances are totally different from those which would be necessary were the Looss hypothesis the correct one.

### The Practical Lessons.

That this is so is well shown in Lieut.-Colonel Leiper's two summaries of conclusions for prevention, one based on Looss' views, and the other on his own. According to Looss all transient collections of water, such as those resulting from showers of rain, road waterings, and domestic waste, are dangerous if freshly contaminated: whereas on Leiper's view they are quite harmless, whether freshly contaminated or not. According to Looss, too, large bodies of water, such as the Nile, canals, marshes, and birkets, are little liable to be infective; according to Leiper they are poten-

* *Journal of the R.A.M.C.*, July, August, September, 1915.

tially very dangerous if inhabited by certain species of snail. All water in a given area, on the Looss hypothesis, would automatically become safe in thirty hours if the native infected population were removed; whereas, on the contrary, Leiper holds that many months would be required before the water would cease to be infective. Then, again, if Looss is correct, infected troops will be liable to reinfect themselves, to spread the disease among other troops and to convey it to any part of the world; whereas the truth, if he is wrong, is that they cannot reinfect themselves or spread the disease except where the proper kinds of snail are found. Looss holds that infection takes place only through the skin; Leiper that it takes place through both the mouth and the skin. Infection, says the former, is due to contact with recently contaminated moist earth or water; the latter holds that it is conveyed by unfiltered water, not recently but at some long time previously contaminated. Lastly, Looss' thesis means that eradication depends on education and complete sanitary control throughout the country: the sustained co-operation of the infected individual is essential. The new researches mean that eradication of the disease can be effected without the co-operation of the infected individual (which it is impossible to obtain), by destroying the molluscan intermediaries.

It is obvious that these researches are of the very first importance, and that Lieut.-Colonel Leiper deserves very well of his comrades no less than of civilisation at large. His paper, at the moment of writing, is not yet complete; and its later instalments will be watched for with great interest. We congratulate him and the Army on a very notable achievement indeed; and as evidence of the thoroughness of his work we may remark that he publishes a bibliography on the subject of bilharzia disease which includes no fewer than 532 references.

